# Leveraging machine learning approaches for predicting potential Lyme disease cases and incidence rates in the United States using Twitter

**DOI:** 10.1186/s12911-023-02315-z

**Published:** 2023-10-16

**Authors:** Srikanth Boligarla, Elda Kokoè Elolo Laison, Jiaxin Li, Raja Mahadevan, Austen Ng, Yangming Lin, Mamadou Yamar Thioub, Bruce Huang, Mohamed Hamza Ibrahim, Bouchra Nasri

**Affiliations:** 1 Harvard Extension School, Harvard University, Cambridge, USA; 2 Department of Social and Preventive Medicine, École de Santé Publique, University of Montreal, Montréal, Canada; 3 Department of Decision Sciences, HEC Montréal, Montréal, Canada; 4 Department of Mathematics, Faculty of Science, Zagazig University, Zagazig, Egypt

**Keywords:** Lyme disease, Twitter, BERT, Incidence Rate

## Abstract

**Background:**

Lyme disease is one of the most commonly reported infectious diseases in the United States (US), accounting for more than $90\%$ of all vector-borne diseases in North America.

**Objective:**

In this paper, self-reported tweets on Twitter were analyzed in order to predict potential Lyme disease cases and accurately assess incidence rates in the US.

**Methods:**

The study was done in three stages: (1) Approximately 1.3 million tweets were collected and pre-processed to extract the most relevant Lyme disease tweets with geolocations. A subset of tweets were semi-automatically labelled as relevant or irrelevant to Lyme disease using a set of precise keywords, and the remaining portion were manually labelled, yielding a curated labelled dataset of 77, 500 tweets. (2) This labelled data set was used to train, validate, and test various combinations of NLP word embedding methods and prominent ML classification models, such as TF-IDF and logistic regression, Word2vec and XGboost, and BERTweet, among others, to identify potential Lyme disease tweets. (3) Lastly, the presence of spatio-temporal patterns in the US over a 10-year period were studied.

**Results:**

Preliminary results showed that BERTweet outperformed all tested NLP classifiers for identifying Lyme disease tweets, achieving the highest classification accuracy and F1-score of $90\%$. There was also a consistent pattern indicating that the West and Northeast regions of the US had a higher tweet rate over time.

**Conclusions:**

We focused on the less-studied problem of using Twitter data as a surveillance tool for Lyme disease in the US. Several crucial findings have emerged from the study. First, there is a fairly strong correlation between classified tweet counts and Lyme disease counts, with both following similar trends. Second, in 2015 and early 2016, the social media network like Twitter was essential in raising popular awareness of Lyme disease. Third, counties with a high incidence rate were not necessarily related with a high tweet rate, and vice versa. Fourth, BERTweet can be used as a reliable NLP classifier for detecting relevant Lyme disease tweets.

**Supplementary Information:**

The online version contains supplementary material available at 10.1186/s12911-023-02315-z.

## Background

Lyme disease has been one of the most reported infectious diseases in the United States (US) and accounts for over $90\%$ of all vector-borne diseases in North America [1–4]. Since 1991 Lyme disease has become an officially notifiable disease in the US and is reported to the Centers for Disease Control and Prevention (CDC). A study trying to evaluate the economic burden of Lyme disease in some high-incidence states within the US estimated the annual cost to be nearly 1 billion US dollars, not including suspected, undiagnosed, or nonacute cases [5, 6].

Lyme disease is a vector-borne disease caused by infection with the bacterium Borrelia burgdorferi [7, 8]. The vector responsible for transmitting to humans is Ixodes-type ticks [9, 10]. Historically, the distribution of ticks in North America was limited to the eastern United States. Ever since then, the tick population has increased and has spread to Southern Canada, where they are increasingly gaining ground [11–13]. The type of ticks differs depending on the geographical location. Due to global warming, North America will be facing more and more warm winters. Since ticks are poikilothermic, with the life stages of each species requiring specific sets of environmental conditions for successful development and survival, they are sensitive for ambient abiotic conditions [14–16]. So, it is normal to expect an increase in the distribution of ticks and the incidence of tickborne diseases such as Lyme disease [12, 17, 18]. In North America, there are two different species of ticks that have been proven to transmit Lyme disease, and their prevalence varies depending on the location. The most predominate specie of ticks is the Ixodes scapularis species, also called deer ticks or black-legged ticks, is found in Northeast and North Central of the US, as well as the Central and Eastern parts of Canada [19, 20]. The second most common is Ixodes pacificus ssp, which is mostly found in the western United States and British Columbia, Canada [9, 21–24]. Lyme disease symptoms are extensive, with typically some dermatological, musculoskeletal, neurological, and cardiac manifestations, but the hallmark of the disease clinical manifestation is a target shaped erythema migrans known as “bull’s-eye” rash, which appears where the tick has bitten in $70-80\%$ of cases [7, 25–28]. This itching can be accompanied by extreme fatigue, headaches, muscle weakness, or dizziness within 30 days of the bite [8, 24, 29]. Without an early and appropriate antibiotic treatment, the symptoms can be persistent for an unknown period and require more complex therapy [4]. However, even with proper antibiotic treatment, $20\%$ of the cases will transit to the late-stage infection called the chronic phase [30–33]. It has been reported that the chronic phase symptoms vary depending on geographical regions, with Lyme arthritis being the most common form in the US [23]. The age distribution of Lyme disease in the US is typically bimodal, with peaks for 5-15 years old and 45-55 years old [7, 26, 29]. Lyme borreliosis infection rates appear to be higher among men under 60 years of age, but otherwise are equal between the sexes, with most cases occurring in June and July [31, 34, 35].

According to CDC, each year in the US, there are approximately 30,000 Lyme disease cases reported through the Nationally Notifiable Disease Surveillance System (NNDSS) by local and health departments all over the country. In particular, 34, 945 confirmed and probable cases of Lyme disease were reported in 2019, which is about $4\%$ more than in 2018 (cf. CDC 2021a and [36]). This surveillance system relies on healthcare workers to report cases, thus only cases who seek medical care and the ones that are being submitted will be reported. Also, Lyme disease cases are often under reported due to difficulties in the diagnostic, therefore the improvement of the CDC surveillance system is essential for timely diagnosis of Lyme disease. Various studies using other methods of surveillance suggest that these numbers are underestimating the actual number of Lyme disease cases in the US since the current surveillance system fails to capture, classify accurately and report in near real-time exactly the counts of Lyme disease cases [2, 6, 14, 37–46]. A recent study, using data from insurance coverage, has estimated that 476, 000 people may get Lyme disease each year in the US [45]. However, another study has suggested an over diagnoses in some high endemicity areas [47].

According to Google Health Vice President David Feinberg: “an estimated $7\%$ of Google’s daily searches are health-related” (The Telegraph, 2019). As the internet has become an essential source of health-related information, it has been suggested that online web-based data seeking information about cases and behaviors can be used as a complementary measure of disease surveillance in the context of public health. Increased digital engagement in the public square of social media has coincided with the rise of a field of study known as “infoveillance,” which can be defined as an analysis of search engine queries to be used as a tool for public health surveillance and policy development [48]. The problem with using web-based data is the complexity of the data collection, which normally goes through keyword extraction. Many studies have proposed various approaches, but most of them rely on three steps: selecting and filtering information, data processing, geocoding of information if necessary, and applying statistical methods to detect trends and predict outcomes [49].

The surveillance of epidemics or infectious disease outbreaks using web-based search data (also known as digital surveillance) has been a recurring topic in the literature in recent years [50]. Several researchers have been interested in monitoring vector-borne diseases using web-based and social media data [51]. Online Google search traffic data helps in the analysis of data related to health topics and the prediction of disease occurrence and outbreaks by providing valuable insights into disease patterns and population behavior [52]. It has been suggested that data generated by web searches can enhance surveillance systems by providing real-time proxy data, thus enabling faster responses. The surveillance of influenza outbreaks was a well-known example of the use of web-based data in health [53]. Various researchers have presented methods to optimize such data for various infectious diseases to help public health agencies and improve the existing surveillance systems. The most famous example is Google flu, which is a Google trend for flu-related web searches.

Another way to use internet data as a surveillance tool is through social media platforms like Twitter or Reddit. As of January 2022, the social microblogging network Twitter has 76.9 million users (approximately twice the population of California), making the United States the first country with most users, where $38.4\%$ of users being female. Also, $47\%$ of US’s internet users aged 15 to 25 used Twitter, compared to only $26\%$ of those aged 56 or higher [54]. With an estimated 500 million tweets registered daily in real time, one advantage of Twitter is that tweets are mostly public, where they can easily be geotagged to a specific location [51]. Among these millions of tweets available daily, only a reduced proportion can be collected free of charge through Twitter APIs (Application Programming Interfaces). However, continuous polling through Twitter APIs can give access to a large dataset allowing detailed analysis [51, 55, 56]. Researchers have since developed various methodologies for collecting and filtering tweets to accurately identify health-related tweets and demonstrate their correlation with national reports of infectious diseases [57].

In accordance with a systematic review of the literature, few studies use internet searches or social media data to assess the distribution of Lyme disease [58]. Google Trends, Twitter, YouTube and discussions from various forums offer an exciting tool for monitoring public attention to specific infectious diseases such as Lyme disease [59–69]. As described previously, Google engine searches with terms associated with Lyme disease show similar patterns in temporal and spatial variations consistent with the trend observed in epidemiological data [63, 66]. Data from social networks such as Twitter also play a role in extending traditional epidemiological models and search by sharing spatial and temporal patterns similitudes [68]. However, most studies using web-based data and social media datasets used a limited set of keywords, which has been inconsistent throughout the literature [59–62, 64–66, 68, 69].

Based on the literature there are numerous keywords that researchers associated with Lyme disease [59–65, 68, 69]. To name a few, they used keywords like Tick, mice, Lyme, Lyme disease, borreliosis, tick bite, cough, borreliosis symptoms, tick sting, circular rash, borrelia burgdorferi sensu lato, bull eye’s pattern rash, and borreliosis. However, this limited diversity of keywords chosen could explain the disparity in the results of studies that attempted to test or prove the performance of models developed from online data activities. As a result, there is a need to emphasize a better method for extracting keywords associated with Lyme disease from these sources. In the US, however, researchers have tried to combine web-based data with the traditional surveillance system to improve the prediction [61, 64, 66]. Among the few studies that have used web-based data from social media and Google trends, the majority of them have used a machine-learned classifier to search for potential and most representative keywords related to the research topic from data collected using a Natural Language Process (NLP).

Surprisingly, no study has used Twitter data as a surveillance tool for Lyme disease in the US. Our study aims to explore the usefulness of Twitter data as a potential surveillance tool for Lyme disease in the US. The main contribution of this paper is as follows:

- Provide a large, curated dataset of over 77, 500 labelled Lyme disease tweets in the US between 2010 and 2019. With a great need to consolidate new data sources relevant to the Lyme disease epidemic, this dataset is publicly available for academics to use in a range of epidemiological research studies;
- Study the empirical performance of a set of state-of-the art NLP-based classifiers for predicting tweets about Lyme disease. In particular, BERTweet can be a reliable and accurate model for identifying potential Lyme disease cases using self-reported tweets;
- Analyze twitter data for describing Lyme disease incidence rates in the US using a large set of accurate Lyme-related keywords. Specifically, explore the spatio-temporal patterns between the tweet counts and disease incidence rates from the CDC.

## Methods

To lay the groundwork for this study, the problem of identifying tweets relevant to Lyme disease is defined as a binary classification task. Given a sample of *n* tweets $\mathcal {D}=\{(x_i,y_i)\}_{i=1}^n$, with $x_i \in \mathbb {R}^d$ is the numerical (or feature vector) representation of the *i*-th tweet and $y_i \in \{0,1\}$ is its class label, where 0 and 1 denote the two classes involving irrelevant and relevant Lyme disease tweets, respectively. We aim to use $\mathcal {D}$ to train, validate, and test an NLP-based classification model so that, given new unknown tweet $x_j$, the learned model can identify whether $x_j$ is a Lyme disease tweet or not, i.e., predict $x_j$’s corresponding class label.

### Data collection and preprocessing

Twitter data was collected using the twitter application program interface (API) with an academic research account to create the training and testing data sets for the binary classification of tweets about Lyme disease. By querying with “$\#\text {lymedisease}$”, “$\#\text {lyme}$”, and “lyme” keywords from the years 2010 to 2019, removing re-tweets, and restricting the language to English only, approximately 1.3 million tweets were collected, with 725, 000 tweets of which originated in the US. GeoPy, a python library, was then used to refine the county and state geolocations of the 725,000 tweets. GeoPy approximates the tweet’s geolocation using the user profile’s location information through third-party geocoders and other data sources. In addition to handling precise locations, GeoPy also utilizes fuzzy matching to infer the meaning of misspelled or abbreviated words such as BKLYN (Brooklyn, NY). This results in obtaining 419, 000 tweets with identifiable geolocations.

A set of keywords frequently associated with Lyme disease was first developed, which was then treated as a set of regular expressions (regex) and applied to label the tweets as either relevant or irrelevant to Lyme disease for the training dataset. Four annotators labeled the data, and then they cross-checked a sample of each other’s work. They first started with 100 tweets each and reviewed all the tweets together, and agreed on how to label the rest of the tweets. Tweets relevant to Lyme disease are labelled with a ‘1’ while the irrelevant ones are labelled with a ‘0’. As shown in Table 1, the regular expression keywords were chosen in certain cases by inspecting the content and analyzing the word frequency in all the collected tweets. In other cases, the keywords were selected based on known medical symptoms or modes of acquiring Lyme disease. For example, the following (slightly modified) tweets were labelled as Lyme disease-related: “My aunt is really sick, we think it‘s Lyme”, “Scientists discovered new Lyme treatment”, “I had Lyme 5 years ago”, “He has high fever and tick bite”, and “Please join us for raising Lyme awareness”. Lastly, the accuracy of labelling tweets using regex in an automated manner with Python will not generate $100\%$ accurately labelled tweets. Therefore, the test/validation dataset was manually labelled by the authors using Table 1’s regular expressions as well as reading the context of the tweet to ensure the test/validation dataset is labelled accurately.

**Table 1:** The set of Keywords used to identify and label tweets relevant to Lyme disease

Keyword Category	Keywords
Mode of Acquiring Lyme Disease	‘hiking’, ‘hike’, ‘forest’, ‘tick’, ‘ticks’, ‘bite’, ‘deer’, ‘deertick’, ‘tickborne’
Symptoms and Medical/Scientific Terms	‘borreliosis’, ‘zoonotic’, ‘infection’, ‘erythema’, ‘migrans’, ‘carditis’, ‘neuroborreliosis’, ‘borrelia’, ‘bacterium’, ‘ixodes’, ‘blackleg’, ‘blacklegged’, ’burgdorferi’, ‘borrelial’, ‘lymphocytoma’, ‘arthritis’, ‘fever’, ‘headache’, ‘headaches’, ‘paralysis’, ‘hearing’, ‘rash’, ‘fatigue’, ‘swollen’, ‘lymph’, ‘chill’, ‘chills’, ‘flu’, ‘sweat’, ‘inflammatory’, ‘inflammation’, ‘neck’, ‘knee’, ‘knees’, ‘stiffness’, ‘heart’, ‘palpitations’, ‘numbness’, ‘tingling’, ‘nausea’, ‘vomiting’, ‘neurologic’, ‘vertigo’, ‘dizziness’, ‘sleepless’, ‘fogginess’, ‘nerve’, ‘irritability’, ‘joint’, ‘depression’, ‘memory’, ‘malaise’, ‘neuro’, ‘long-haul’, ‘long haul’, ‘neurologist’, ‘dermatologist’, ‘late stage’, ‘early stage’, ‘antibiotic’, ‘specialist’, ‘lyme disease’, ‘lymedisease’, ‘physician’, ‘doctor’, ‘symptom’, ‘ache’, ‘pain’, ‘diagnose’, ‘diagnosis’, ‘patient’, ‘hospital’, ‘clinic’, ‘cure’, ‘treat’, ‘heal’, ‘disease’, ‘medication’, ‘medicine’, ‘therapy’, ‘infection’, ‘lyme\’s’, ‘tested positive’, ‘tested negative’, ‘lyme test’, ‘lyme\’s test’, ‘medical care’, ‘med check’, ‘medical checkup’, ‘meds’, ‘health’, ‘illness’, ‘bulls eye’, ‘bulls-eye’, ‘bullseye’, ‘bull\’s eye’, ‘bull\’s-eye’, ‘death’, ‘die’, ‘red color’
Common Vernacular/Colloquial Phrases	‘have lyme’, ‘had lyme’, ‘having lyme’, ‘has lyme’, ‘get lyme’, ‘gets lyme’, ‘got lyme’, ‘getting lyme’

**Train Dataset Preparation:** Since the primary goal is to build NLP models that can capture the context and content of tweets about Lyme disease rather than geolocations, portions of the 306, 000 tweets without geolocation were considered for the training process. Specifically, tweets were randomly selected from 2010 to 2019 years to train NLP classification models to recognize differences in communication style that can occur over time. Exactly, 35, 000 geolocated tweets. were selected from each year at random and labelled as relevant to Lyme disease using the keywords stated in Table 1. Furthermore, 25, 000 tweets (i.e., 2,500 from each year for a total of ten years) were randomly selected and labelled as irrelevant to Lyme disease based on the same set of keywords. For example, the tweet topic could be related to the city Lyme rather than Lyme disease. Finally, 10, 000 neutral tweets were included as irrelevant to Lyme disease. The neutral tweets are those that do not contain the word ”Lyme” and are about unrelated topics to Lyme disease. From a technical standpoint, neutral tweets are essential to improve the performance of NLP classification models in identifying random tweets. As a result, the training dataset contained 70,000 tweets in total: 35,000 tweets about Lyme disease and 35,000 tweets (i.e., 25,000 + 10,000) that are not about Lyme disease.

**Test/Validation Dataset Preparation:** To create the test and validation datasets (ground truth), 6, 000 geolocated tweets were randomly selected from the 419,000 tweets, and then manually labelled based on each tweet’s content. Of the 6,000 tweets, 3,800 and 2,200 were labelled as relevant and irrelevant to Lyme disease respectively. Finally, an additional 1,500 neutral tweets were labelled as irrelevant to Lyme disease. In total, 7,500 tweets were collected for the purpose of validating and testing the NLP models. In particular, 3,500 tweets were used in the validation step and the remaining 4,000 tweets served as testing data.

Since there is a considerable need to combine new data sources related to the Lyme disease outbreak, we offer the training and testing datasets openly available to academics for use in a variety of epidemiological research investigations.

### Identifying Lyme disease-related tweets

Several NLP-based classification models were built and evaluated for classifying the tweets as Lyme disease related. Since all of these classifiers are frequently trained to understand contextualized representations and semantics of tweets, the training and the testing datasets must be further processed into a sequence of lemmatized tokens, each of which is represented as a numerical feature vector (namely word embedding). Specifically, stop words and special characters (i.e., hashtags) were first removed. Then, a set of word embedding methods was used to tokenize and lemmatize the tweets, which were then fed into the classification models. The following embedding-classification model combinations were evaluated:

**Word2vec** [70] **and XGBoost** [71]. Word embedding is a common method for representing words in a high-dimensional space based on their similarity. Gensim [72] was used to train a model to extract the word embedding of a given corpus (i.e., collection of documents). Gensim also has built-in embeddings trained on large corpora that can be fit into the given corpus. In particular, the obtained Gensim Word2vec vector representations of all the documents in the corpus were fed into XGBoost for classification.

**TF-IDF** [73] **and Logistic Regression** [74]. TF-IDF, which stands for Term Frequency-Inverse Document Frequency, was used to obtain tweet embeddings. TF-IDF is one of the more reliable vectorization methods. Its covariate matrix consists of the weight (i.e., TF-IDF score) of each word, which is upgraded by how frequently the word appears in each tweet but downgraded by how often it appears in the entire corpus. Therefore, the weight of each word in the matrix is balanced so that the importance of a given word is appropriately emphasized when training the logistic regression model for classification purpose. For this study, the TF-IDF embeddings of the tweets were fed into the logistic regression model for classification.

**BERT** [75]. BERT, which stands for a Bidirectional Encoder Representations from Transformers, is a transformer-based language model that has been pre-trained on Wikipedia and the Brown Corpus. BERT model learns language embeddings through two tasks: mask language modelling and next sentence prediction. A BERT model was further trained and fine-tuned to classify Lyme disease-related tweets based on the context of the tweets. The cleaned tweets were then vectorized using BERT tokenizer, which uses the WordPiece tokenization technique. For example, the word “spreading” is tokenized into “spread” and “$\#\#\text {ing}$” such that similar contextual embeddings can be drawn from different samples with different word formats.

**BERTweet** [76] is a large-scale pre-trained model for English Tweets. It was released in 2020 and had similar architecture as BERT base. It is trained using the RoBERTa pre-training procedure on an 80 GB corpus of 850 million tweets. The model has been proven to be effective in named-entity recognition, part-of-speech tagging, and text classification.

For the purpose of training, validating, and testing the models, the tweets were cleaned by removing Hashtags, URL links, and username. This reduces the noise withing the tweets themselves. Profane words were removed in the tweets before building the tokens. We used the NLP library called ’ProfanityFilter’ to censor the content. Also, emojis were removed from the tweets to improve the tokenization process.

To guarantee a fair comparison, the same train and test datasets were used to build all the NLP classification models. For the XGBoost classifier model, hyper-parameters of a learning rate of 0.01 and a number of estimators of 100 were used. The logistic regression model was trained with and without $7-$fold cross-validation after $\text {TF-IDF}$ vectorization. In addition, various hyper-parameters such as $\text {L1}/\text {L2}$ regularization, inverse of regularization strength values ranging from 0.1 to 100, and solvers such as ‘liblinear’, ‘lbfgs’, ‘sag’, and ‘saga’ were evaluated.

To configure the BERT and BERTweet models, Adam algorithm was chosen as the optimizer for its good performance in handling sparse data like short tweets and low sensitivity to learning rate. Adam algorithm was needed to handle potential issues of sparse vectorized texts due to the length limitation on the nature of tweets. In addition, binary Cross-Entropy was used as the loss function to maximize likelihood estimation while Sigmoid was used as the activation function to better suit binary classification problems. A learning rate of $-2 \times 10^{-5}$, a weight decay of 0.01, and a batch size of 64 were used. The training procedure took about 1 hour with ‘accuracy’ as the best metric for an early stopping value of 0.001. The models ran for 2 epochs to avoid overfitting the training dataset. Note that the hyperparameter value ranges used as suggested in [75]. It is recommended to use only 2 to 4 epochs and a batch size of 64. This statement was confirmed by our finding; See Supplementary Table 1 (Additional File 1), for BERTweet. In fact, The validation loss starts to increase after 2 epochs which demonstrates the presence of overfitting. This is because training BERT’s like pre-trained models with a large number of epochs frequently results in catastrophic forgetting, where the model forgets the pre-trained weights.

Consequently, the results of the underlying NLP classifiers used to identify Lyme disease tweets were compared to the keyword-based labelling method (explained in Subsection “Data collection and preprocessing”), which can serve as a good baseline for comparison. Furthermore, the following standard metrics were calculated to assess the accuracy and efficiency of results obtained from all the classifiers on the testing dataset: classification accuracy, F1-score, precision, and recall.

### Descriptive spatio-temporal pattern analysis

We studied the spatio-temporal correlation between tweet counts resulting from the best classification algorithm and CDC disease incidence rates. This is crucial because, if a correlation exists, tweet counts could be used to predict Lyme disease incidence rates. This is because if an outbreak occurs in a specific community, then there will be an increase in tweets about Lyme disease due to increased awareness, which implies that any tweets about Lyme disease can be used to identify Lyme disease cases in real time.

## Results and discussion

### Performance of the NLP classification models

As shown in Table 2, BERTweet outperformed all tested classification models, achieving the highest classification accuracy, F1, precision, and recall scores. BERT showed a decent ability to classify Lyme disease-related tweets, coming slightly behind BERTweet with an F1- score of $89\%$ and a classification accuracy of $90\%$. The $\text {TF-IDF}$ logistic regression model was marginally more accurate than the basic keyword labelling method because there were slightly more false positives (i.e., lower precision score of $94\%$) but fewer false negatives (i.e., higher recall score of $81\%$), resulting in a very similar F1 score of $87\%$ compared to $86\%$ for keyword labelling. In addition, the total number of correctly predicted true positives and true negatives across the entire dataset was comparable to the keyword labelling method. On the contrary, the Word2vec-XGBoost classification model was considerably less accurate than the basic keyword labelling method, producing more false positives (i.e., a lower precision score of $78\%$) and false negatives (i.e., a very low recall score of $73\%$), resulting in a lower F1 score. Furthermore, the total number of correctly predicted true positives and true negatives was less than the keyword labelling method, resulting in lower accuracy score of $76\%$.

**Table 2:** Performance comparison of NLP classification models on the test dataset. The highest score values are shown in bold

NLP Classification Model	Accuracy	F1 Score	Precision	Recall
Keyword-based labelling	0.84	0.86	**0.97**	0.77
Word2vec and XGBoost	0.76	0.75	0.78	0.73
$\text {TF-IDF}$ and Logistic Regression	0.88	0.87	0.94	0.81
BERT	**0.90**	0.89	0.96	0.83
BERTweet	**0.90**	**0.90**	0.95	**0.85**

One of the reasons for the poor performance of the Word2vec-XGBoost is that it is commonly difficult to properly tune the hyperparameters of XGBoost, which frequently results in XGBoost overfitting. Furthermore, due to the large size of the vocabulary, Gensim Word2vec may be difficult to train and require a longer training time to extract the features, particularly when using traditional functions such as the softmax. While approximation algorithms such as negative sampling or hierarchical softmax may be applied to alleviate this issue in Word2vec, the produced word vectors are not distributed uniformly in the vector space, leading to insufficient vector space utilization. Moreover, Word2vec is a static word-based model that extracts content from a corpus and generates context-independent co- embeddings based on occurrence information. It frequently cannot be dynamically optimized and cannot take word position and ordering into account.

$\text {TF-IDF}$, like Word2vec, does not consider context and does not capture the position of the words. It is computationally less intensive than Word2vec, but, unlike Word2vec, $\text {TF-IDF}$ does not take into account semantics and co-occurrence information across documents. Apart from tweet embeddings, we believe that the superior accuracy of $\text {TF-IDF}$ logistic regression over Word2vec-XGboost is due to the dominance of logistic regression over XGboost, which is consistent with the fact that logistic regression models typically have more accurate probability calibration in comparison to gradient boosting approaches (see Niculescu-Mizil, et.al. 2005 for more details). This could also support the hypothesis that our datasets are likely to be linearly separable, because XGboost captures nonlinear relationships while logistic regression fits more linear ones. BERT and BERTweet, on the other hand, are context-dependent models that produce accurate word representations that consider word position and ordering. They can also understand different semantic meanings of words in different tweet contexts. This could explain why pre-trained models like Bert and BERTweet perform better in our results than Gensim word2vec-XGboost and $\text {TF-IDF}$ logistic regression.

### Spatio-temporal pattern analysis

This experiment examines the spatio-temporal correlation between tweet counts and CDC disease incidence rates. Since BERTweet produced the most accurate classification results in Subsection “Performance of the NLP classification models”, this model was chosen to classify the untouched 419, 000 tweets to correlate or predict Lyme disease cases. Afterwards, exploratory data analysis was conducted as follows.

#### CDC Lyme disease counts vs. classified tweet counts

**Fig. 1:**
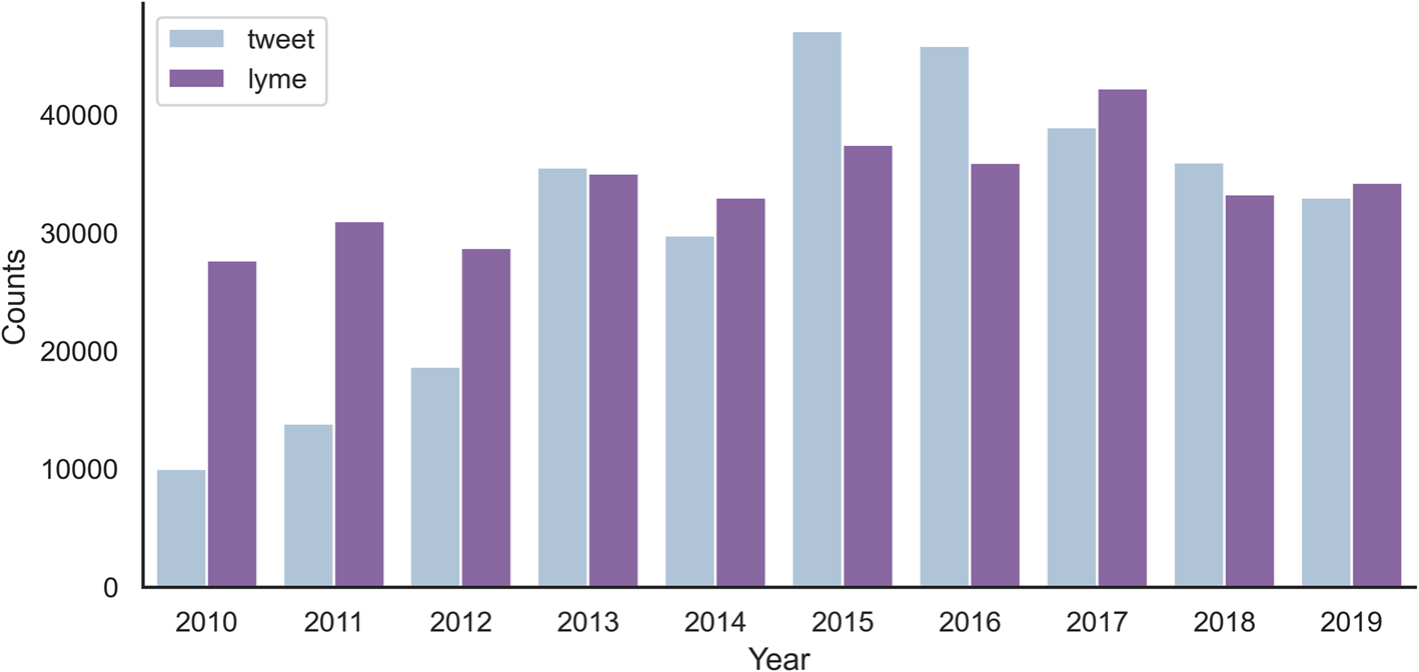
Tweet count and Lyme disease count comparison in the US from 2010 to 2019

**Fig. 2:**
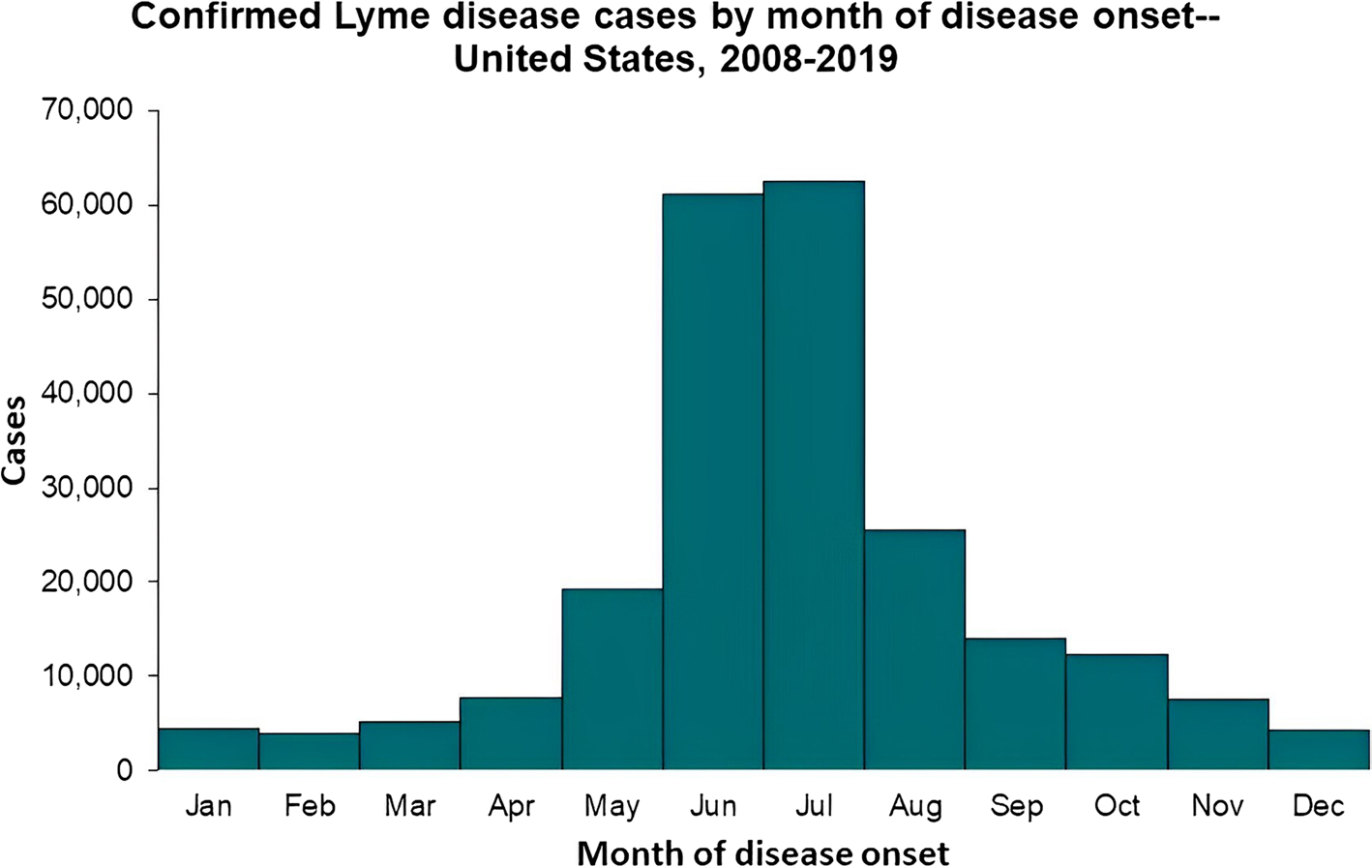
Histogram of confirmed Lyme disease cases in the US between 2010 and 2019

The classified tweet counts were compared to Lyme disease counts between 2010 and 2019 to understand their effective sample sizes. We used the CDC’s Lyme Disease data counts. As shown in Fig. 1, tweet counts and Lyme disease counts followed relatively similar trends with a few exceptions. In particular, the Pearson correlation between the two counts was 0.82, while the Spearman correlation was 0.92, both with p-values less than 0.05. From 2010 to 2012 when Twitter membership and usage were still low, tweet counts were lower and did not correlate to the Lyme disease counts as expected. In contrast, the observed spike in tweet counts between 2015 and 2016 was possibly attributable to the increased popularity of Twitter and general awareness of the disease. Furthermore, Lyme disease cases have a seasonal trend that peaked during the summer months of June and July, as shown in Fig. 2.

**Fig. 3:**
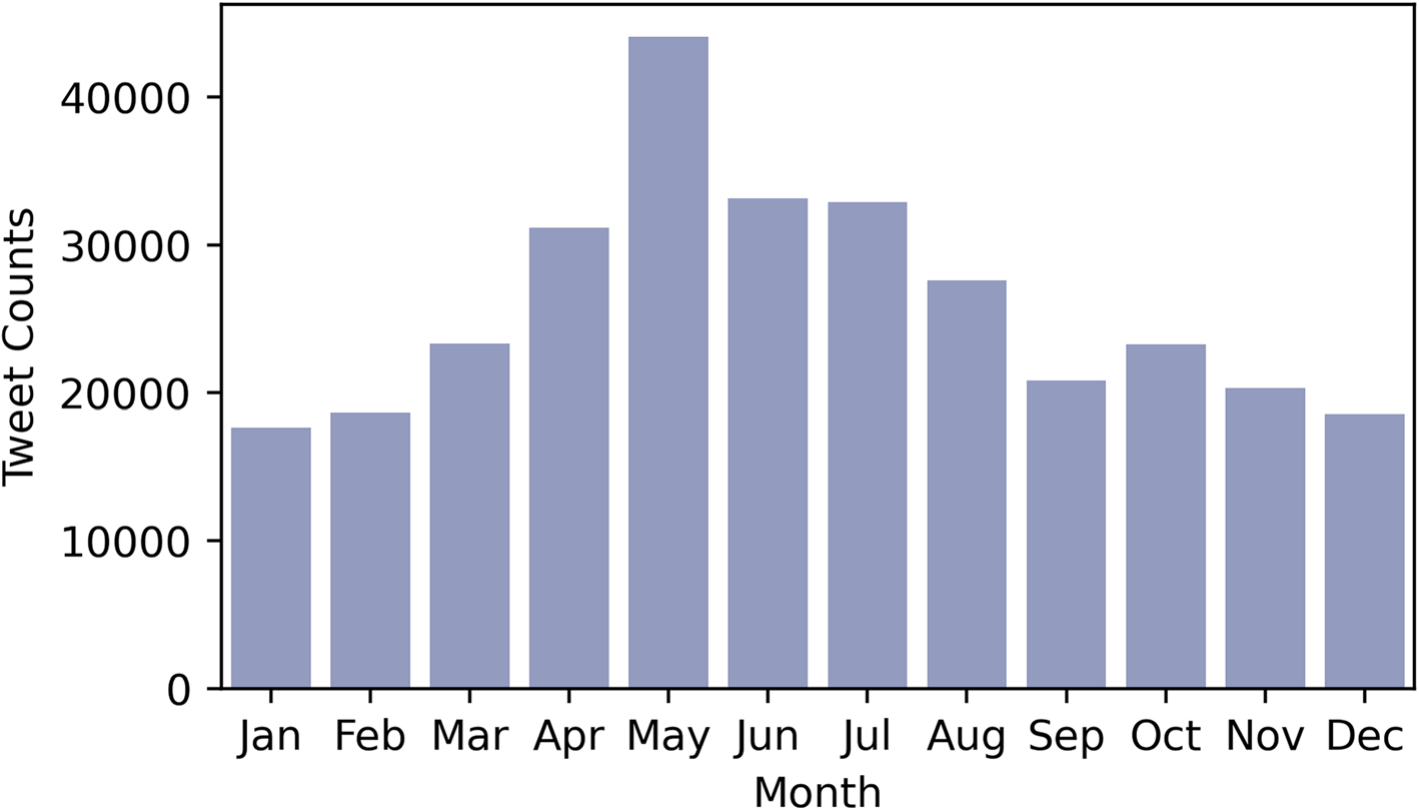
Histogram of Lyme disease tweet counts by month in the US from 2010 to 2019

Tweet counts were also examined to see if similar patterns could be discerned. Figure 3 shows that tweets related to Lyme Disease increased in May, June, and July. In addition, the month of May had the highest peak in tweet counts, which was one month prior to the peak in Lyme disease discussions. This can be explained by three major factors: (1) it can take up to one month for Lyme disease symptoms to appear; (2) Lyme disease can be difficult and time-consuming to diagnose; (3) organizations or Twitter users send out Lyme disease warnings in advance. Thus, if historical monthly case counts were available to build the correlation, Twitter could potentially provide information on how Lyme disease spreads over the course of a year.

#### Tweet rate and Lyme incidence rate on US map

**Fig. 4:**
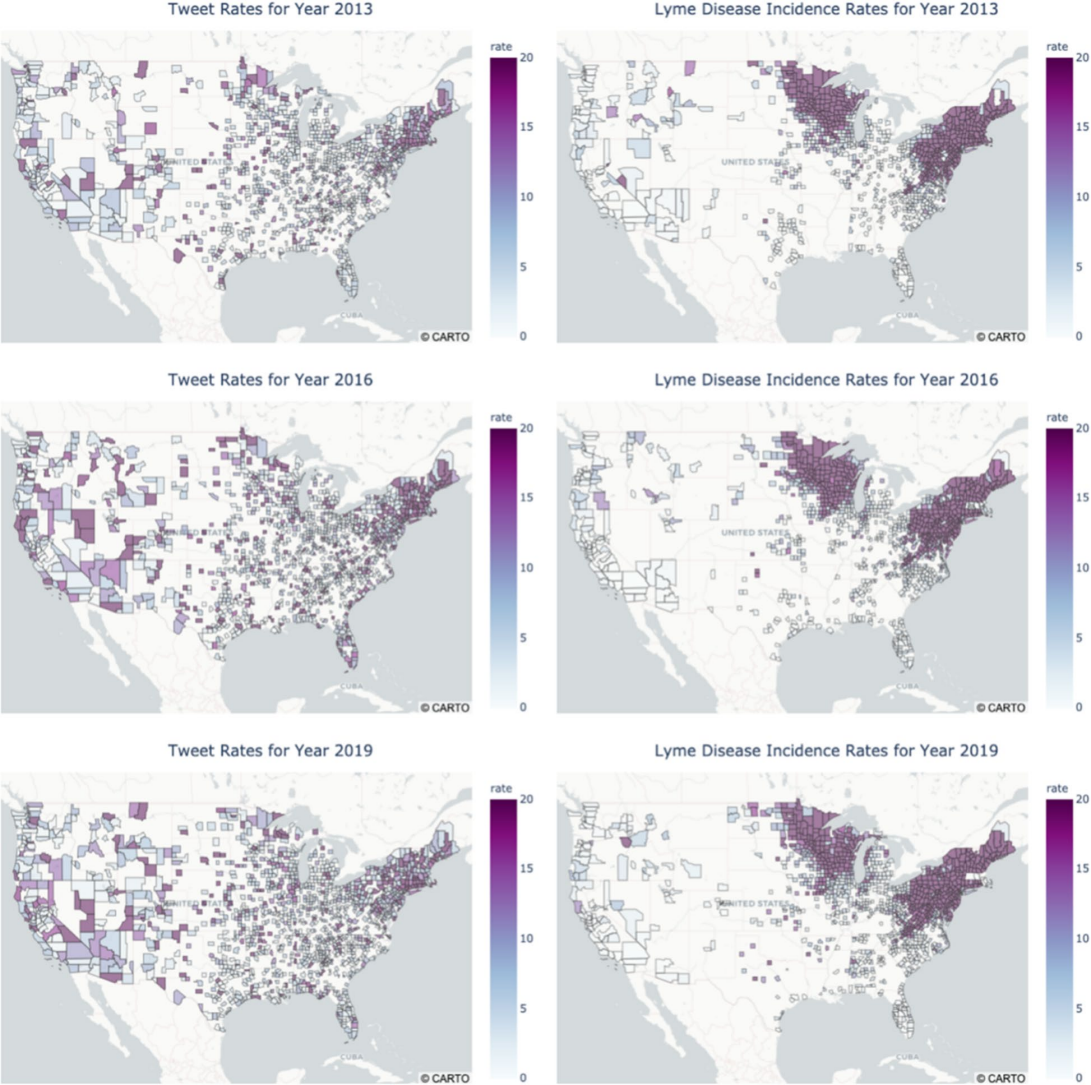
Comparison of selected Geo Maps for Lyme disease tweets and cases in the US (2013, 2016, and 2019). This figure was plotted using Plotly library in Python

To comprehend the patterns of how tweets and Lyme disease cases spread over different regions of the US, a comparison plot of tweet rates (number of cases per 100,000 people) versus Lyme disease incidence rates (number of cases per 100,000 people) for each year was generated. Figure 4 captures the plots for three selected years (2013, 2016, and 2019). For simplicity, only figures for the three most representative years are shown. The preliminary remark is that the distributions of Lyme disease data across years were quite similar. The Northeast, Upper Midwest, and Northwest were the regions with the highest incidence rates. The second observation is that there was a consistent pattern indicating that West and Northeast regions had a higher tweet rate across the years, despite a small increase in total tweets in later years.

Nevertheless, the graphs also revealed that the distributions of Lyme disease incidence and tweet rates were not identical. For many counties, a high incidence rate was not necessarily associated with a high tweet rate or vice versa. For example, it is common for a county with an increased incidence rate to not have a high tweet rate, perhaps due to limited access to the Internet or limited interest in discussing Lyme disease. This indicates that not all counties in the United States have a similar distribution between the two rates, which requires further investigation to understand these discordances.

## Conclusion and future work

This study has focused on the understudied problem of using Twitter data as a surveillance tool for Lyme disease in the US. As such, the novelty of this work lies in analyzing web-based Twitter data for describing Lyme disease incidence rates in the US based on an accurate selection of keywords. To the best of our knowledge, this is the first study to use Twitter data to predict Lyme disease incidence in the US without integrating it with current surveillance systems. Although this study focused on mining Twitter and analyzing data from the US given it is one of the world’s hotspots for Lyme disease, our methodology can be easily generalized to other regions around the world.

We successfully collected approximately 1.3 million tweets, which were then preprocessed to extract the most relevant tweets about Lyme disease, many of which included geolocations. Using a set of keywords filter, these tweets were manually labeled as relevant or irrelevant to Lyme disease. Afterwards, a variety of NLP classification models such as logistic regression, XGBoost, BERTweet, and BERT were trained and tested to classify Lyme disease tweets. Subsequently, spatiotemporal correlations were evaluated between the classified tweet counts obtained from BERTweet and the CDC disease incidence rates in the US.

Furthermore, the study has revealed a few key findings. First, there is a very strong correlation between classified tweet counts and Lyme disease counts, with both following relatively similar trends with some exceptions. Second, in 2015 and early 2016, the social media platform Twitter played a critical role in raising general awareness of Lyme disease. Third, there was a consistent pattern indicating that the West and Northeast regions had a higher tweet rate over time. However, a high number of tweets does not necessarily mean a high number of Lyme cases. We have noticed that in many counties, there are a high number of cases reported on Twitter without observing the same trend from CDC data. This will be explored further in future research. Finally, BERTweet outperformed all tested NLP-based classifiers. Overall, these preliminary results in Experiment I support its accuracy, as BERTweet achieved the highest classification accuracy and F1-score of $90\%$. Moreover, it is worth noting that the study’s primary goal was to demonstrate that leveraging a larger dataset that has been meticulously curated can indeed yield impressive results. While we think the choice between dataset size and manual labelling effort should be dependent on available resources and the specific goals of the application, apparently a much smaller but manually labelled dataset could be a potential efficient solution to handle scenarios where manual labelling is infeasible or there is a limited amount of data.

One limitation of this study is that many of the tweets collected may not be confirmed Lyme disease cases. Our team is working currently to refine the classification of the tweets by identifying and labeling tweets that are truly confirmed and potential Lyme disease cases to obtain more accurate correlations across all US counties and build an early warning system for Lyme disease in the US. Another limitation to address in our future studies is to avoid neutral tweets or combine them with non-lyme disease tweets.

### Supplementary Information

**Additional file 1.**

## Data Availability

The datasets analyzed, and the code used to support the conclusions of this study are available on the GitHub repository at the following link https://github.com/csci-e597-sp2022-lymedisease/lymedisease.

## Abbreviations

CDC: Centers for Disease Control and Prevention
TF-IDF: Term frequency-inverse document frequency
XGBoost: Extreme gradient boosting
BERT: Bidirectional Encoder Representations from Transformers
BERTweet: Bidirectional Encoder Representations from Transformers for English Tweets
NLP: Natural Language Processing
ML: Machine learning
Regex: Regular expressions
US: United states
