# Supplementary material for: Leveraging machine learning approaches for predicting potential Lyme disease cases and incidence rates in the United States using Twitter
Source: BMC Med Inform Decis Mak. 2023 Oct 16;23:217. doi: 10.1186/s12911-023-02315-z (PMC10578027; doi:10.1186/s12911-023-02315-z)
Supplement: Supplementary file 1 — Additional file 1. [file 12911_2023_2315_MOESM1_ESM.pdf]

Supplementary Table 1

Training and Validation loss by epochs for the BERTweet model-based 70,000 tweets

Epoch	Training Loss	Validation Loss
1	0.1974	0.1256
2	0.1140	0.1123
3	0.0852	0.1239
4	0.0491	0.1456
5	0.0344	0.1670
6	0.0258	0.1853
7	0.0120	0.1959
8	0.007	0.2022
9	0.0082	0.2061
10	0.0030	0.2110
